# COVID-19 lockdown and mental health: why we must look into oncology units

**DOI:** 10.1017/S0033291720002500

**Published:** 2020-07-03

**Authors:** Carolyne Croizier, Jean-Baptiste Bouillon-Minois, Jacques-Olivier Bay, Frédéric Dutheil

**Affiliations:** 1 Service thérapie cellulaire et hématologie clinique, CHU Clermont-Ferrand, Université Clermont Auvergne, Clermont-Ferrand, France; 2 CNRS, LaPSCo, Physiological and Psychosocial Stress, University Hospital of Clermont-Ferrand, Emergency Medicine, Clermont Auvergne University, Clermont-Ferrand, France; 3 CNRS, LaPSCo, Physiological and Psychosocial Stress, University Hospital of Clermont-Ferrand, Occupational and Environmental Medicine, WittyFit, Clermont Auvergne University, Clermont-Ferrand, Auvergne-Rhône-Alpes, France

## To the editor:

We read with interest the article by Lazarov et al. (2019) in *Psychological Medicine* which demonstrates that ‘*depressive and posttraumatic symptoms constitute two separate diagnostic entities, but with meaningful between-disorder connections, suggesting two mutually-influential systems*’. Post-traumatic stress disorder (PTSD) is a mental health condition that is triggered by a terrifying event − either experiencing it or witnessing it. Symptoms may include flashbacks, nightmares and severe anxiety, as well as uncontrollable thoughts about the event. They get worse, for months or even years, and finally interfere with day-to-day functioning.

With Corona Virus Disease 19 (COVID-19), more than 3.5 billion people globally were under lockdown. The local epidemic that initially appeared in Wuhan, Hubei, China (Chan et al., 2020) quickly became a global pandemic that forced worldwide governments to enact quarantine status and massive lockdown, in an effort to contain the propagation of the virus (Gostin, Hodge, & Wiley, 2020).

In oncology, especially in clinical hematology units, lockdown has been a common practice for years. When a diagnosis of acute leukemia requires a rapid intensive induction chemotherapy, a patient can get locked in a cleanroom for a long period (4−6 weeks) to prevent infectious complications of aplasia (Döhner et al., 2017). An allogenic or autologous hematopoietic stem cell transplantation (HSCT) can require a long hospitalization for the same reasons, although patients in this situation are more prepared to these strict conditions of treatment. These treatments require cleanroom and strict hygienic measures, such as regularly washing one's hands with an alcohol-based hand rub, wearing a mask, maintaining social distancing during visits, or limiting visits to one per day (Holý & Matoušková, 2012). These measures invade the everyday of patients.

Both meant to prevent infections, lockdown measures in oncology and in the COVID-19 world therefore have close durations and measures. But, a sudden lockdown cannot be done without psychological repercussions, and studies have already been done to know the psychological consequences in oncology. For acute leukemia patients, stress disorders and PTSD are common. They have been proven to be associated with the level of pain, the quality of the relationships with health-care providers, and other individual psychological characteristics of the patients (Rodin et al., 2018). Mental consequences are crucial issues for patients who have gone through an HSCT or induction for acute leukemia, certainly due to lockdown. Six months after an HSCT, a significant proportion of patients meet criteria for PTSD and depression (El-Jawahri et al., 2016), which seems logical in the view of conclusions derived by Lazarov et al. (2019).

Psychological consequences of COVID-19 quarantine are already studied and PTSD is obviously mentioned (Brooks et al., 2020). Several risk factors are implicated in the development of these psychological disorders but the ‘lockdown’ factor seems to be important. In this regard, mental health studies on patients who have been treated in oncology may very well be of use to approach the global impacts of lockdown measures on half the world's population's health condition.
